# Sex-, Age-, and Projection-Related Variability in Pneumothorax and Pulmonary Mass Detection Frequencies on Chest Radiographs: A Retrospective Cross-Sectional Study

**DOI:** 10.3390/diseases14070257

**Published:** 2026-07-16

**Authors:** Josef Yayan

**Affiliations:** Department of Internal Medicine, Division of Pulmonary, Allergy, and Sleep Medicine, Helios University Hospital Wuppertal, Witten/Herdecke University, Heusnerstr. 40, 42283 Wuppertal, Germany; josef.yayan@hotmail.com; Tel.: +49-0202-896-3936; Fax: +49-0202-896-3901

**Keywords:** pneumothorax, pulmonary mass, chest radiography, epidemiology, diagnostic imaging, radiographic projection, age differences, sex differences, respiratory diseases, thoracic imaging

## Abstract

Background: Chest radiography remains one of the most widely used imaging modalities for the detection of thoracic abnormalities, including pneumothorax and pulmonary masses. However, the frequency with which these findings are identified may vary according to demographic characteristics and radiographic acquisition techniques. Understanding such variability is important for interpreting large imaging datasets and may improve diagnostic assessment and epidemiological research. Therefore, this study aimed to evaluate sex-, age-, and projection-related differences in the dataset-derived detection frequencies of pneumothorax and pulmonary mass annotations in a large chest radiograph dataset. Methods: A retrospective cross-sectional analysis of 112,120 frontal chest radiographs obtained from 30,805 adult patients in the NIH ChestX-ray14 dataset was performed. Only anteroposterior (AP) and posteroanterior (PA) projections were included. Patients younger than 18 years, radiographs lacking demographic metadata, and lateral projections were excluded. Pneumothorax and pulmonary mass annotations were defined according to the corresponding NIH ChestX-ray14 labels generated from radiology reports using natural language processing. Patients were stratified according to sex and age group. Statistical analyses included chi-square tests, adjusted odds ratios (adjusted ORs), image-level multivariable logistic regression analyses, and corresponding patient-level generalized estimating equation (GEE) analyses to account for repeated radiographs from the same patient. Sensitivity analyses, including simulated annotation misclassification scenarios, were conducted to evaluate the robustness of the observed associations. Results: A total of 4988 pneumothorax and 5588 pulmonary mass annotations were identified. Pneumothorax detection frequencies were significantly higher among females aged 41–80 years (adjusted OR range: 0.66–0.68; *p* < 0.0001), whereas pulmonary mass annotations were more frequently detected in males, particularly in individuals aged 21–40 years (adjusted OR 1.52; *p* < 0.0001). Female patients were older on average and underwent PA imaging more frequently than male patients. AP projection was associated with lower pneumothorax annotation detection frequencies, whereas its association with pulmonary mass annotation detection was attenuated after patient-level analysis. Patient-level GEE analyses confirmed the robustness of the primary image-level findings. Conclusions: Detection frequencies of pneumothorax and pulmonary mass annotations varied systematically according to sex, age, and projection type, demonstrating substantial demographic and technical variability within a large chest radiograph dataset. These findings highlight the importance of considering demographic characteristics and imaging acquisition factors when interpreting large chest radiograph datasets. Because the findings were based on NLP-derived dataset annotations rather than reference-standard diagnoses, they should be interpreted as dataset-level associations rather than estimates of disease prevalence. Improved recognition of demographic and projection-related variability may contribute to more accurate interpretation of chest radiographs and more reliable imaging-based epidemiological research.

## 1. Introduction

Chest radiography remains the most widely used imaging modality worldwide because of its accessibility, low cost, and rapid image acquisition. It plays a central role in the evaluation of a broad range of thoracic diseases and is frequently used in both emergency and routine clinical settings. Among radiographic abnormalities, pneumothorax and pulmonary masses are of particular clinical importance because they may require urgent intervention, additional diagnostic procedures, or long-term clinical follow-up [1,2,3].

Pneumothorax is a potentially life-threatening condition characterized by the presence of air within the pleural space, resulting in partial or complete lung collapse. Its incidence varies according to age, sex, smoking status, and underlying pulmonary disease [4,5,6]. Pulmonary masses also represent clinically significant findings, often requiring further evaluation to exclude malignancy or other serious pulmonary disorders. The prevalence and radiographic presentation of pulmonary masses may also vary across demographic groups and clinical settings [7,8,9].

The interpretation of chest radiographs is influenced not only by disease prevalence but also by patient-related and technical factors. Age and sex are important determinants of many respiratory diseases and may influence both the occurrence and radiographic appearance of thoracic abnormalities [10,11,12]. In addition, image acquisition techniques, particularly anteroposterior (AP) and posteroanterior (PA) projections, can affect lesion visibility and diagnostic confidence. AP radiographs are commonly obtained in critically ill, bedridden, or intensive care patients, whereas PA radiographs are typically performed in ambulatory individuals, introducing potential differences related to patient selection and imaging conditions [13,14].

Large public imaging repositories have created new opportunities to investigate population-level patterns of radiographic findings. The NIH ChestX-ray14 database is among the largest publicly available chest radiograph datasets and has been widely used for studies evaluating thoracic abnormalities across diverse patient populations [15,16]. The dataset contains image-level annotations generated from radiology reports using natural language processing rather than independently verified clinical diagnoses, an important consideration when interpreting observed detection frequencies. Such datasets provide valuable opportunities to explore demographic and acquisition-related variability in radiographic findings at a scale that is difficult to achieve in single-center studies. Despite the widespread use of chest radiography, evidence regarding sex-, age-, and projection-specific differences in the frequency of pneumothorax and pulmonary mass findings remains limited. Most previous studies have focused on disease-specific cohorts or clinical outcomes rather than large-scale radiographic datasets. Therefore, important gaps remain regarding the influence of demographic and technical factors on the distribution of these findings.

Despite the widespread use of large chest radiograph datasets, relatively few investigations have systematically examined how age, sex, and radiographic projection influence the detection frequencies of clinically important findings such as pneumothorax and pulmonary masses. A better understanding of these factors may improve the interpretation of large imaging datasets and contribute to more accurate epidemiological and diagnostic assessments.

To evaluate age-, sex-, and projection-related differences in the detection frequencies of pneumothorax and pulmonary mass annotations within a large chest radiograph dataset. Descriptive analyses, multivariable regression analyses, and sensitivity analyses were performed to characterize demographic and technical determinants of these findings and to provide insights into variability relevant to diagnostic imaging and epidemiological research. Unlike previous studies that primarily focused on disease-specific cohorts or image classification models, the present study systematically evaluates demographic- and projection-related variability in two clinically relevant radiographic findings within one of the largest publicly available chest radiograph datasets while explicitly assessing the influence of repeated examinations on the observed associations.

## 2. Materials and Methods

### 2.1. Data Source and Study Population

This retrospective cross-sectional study was conducted using the publicly available NIH ChestX-ray14 dataset developed by the National Institutes of Health (NIH, Bethesda, MD, USA), which contains 112,120 frontal chest radiographs obtained from 30,805 adult patients. Image-level annotations for thoracic abnormalities were generated from associated radiology reports using natural language processing (NLP). The dataset is one of the largest publicly available repositories of chest radiographs and has been widely used in thoracic imaging research.

Only patients aged 18 years or older were included in the present analysis. Radiographs lacking age or sex metadata, as well as lateral projections, were excluded. Available metadata included patient sex, age, image projection (anteroposterior [AP] or posteroanterior [PA]), patient identifier, and binary annotations for radiographic findings. The patient identifier was used to account for repeated radiographs obtained from the same individual in patient-level statistical analyses. A study flow diagram was constructed to illustrate patient selection and exclusion criteria, including the number of excluded radiographs for each exclusion criterion and the final numbers of included radiographs and unique patients.

### 2.2. Definition of Radiographic Findings

The primary outcomes of interest were pneumothorax and pulmonary mass. These findings were identified using the dataset annotations. In the NIH ChestX-ray14 dataset, “Mass” refers to the corresponding dataset label generated from radiology reports and should not be interpreted as a histopathologically confirmed pulmonary malignancy. Accordingly, pneumothorax and pulmonary mass represent NLP-derived dataset annotations rather than independently verified clinical diagnoses. To minimize variability associated with heterogeneous imaging protocols, only frontal AP and PA radiographs were analyzed. Demographic variables (sex and age) and technical imaging parameters (projection type) were extracted from the dataset metadata. Because the annotations were derived from radiology reports rather than independent image review, the identified findings should be interpreted as dataset-based radiographic annotations rather than definitive clinical diagnoses.

### 2.3. Data Processing and Grouping

Patients were categorized according to sex (male or female) and stratified into five age groups: 18–20 years, 21–40 years, 41–60 years, 61–80 years, and 81 years or older. These age categories were selected to reflect clinically meaningful life stages while maintaining sufficient numbers of radiographs within each subgroup for reliable statistical comparisons. For each subgroup, the frequency of pneumothorax and pulmonary mass annotations was calculated. Mean age, standard deviation, and projection distribution were also summarized. Additional dataset characteristics were presented in a descriptive table to enhance transparency and reproducibility.

### 2.4. Statistical Analysis

Continuous variables were compared using independent-samples *t*-tests, whereas categorical variables were analyzed using chi-square tests. Differences in detection frequencies between sexes across age groups were quantified using adjusted odds ratios (adjusted ORs) with 95% confidence intervals (CIs).

Image-level multivariable logistic regression models were performed to evaluate the independent associations of sex, age, and projection type with pneumothorax and pulmonary mass annotation detection. To account for repeated radiographs from the same patient, corresponding patient-level generalized estimating equation (GEE) logistic regression models were fitted using patient ID as the clustering variable. An exchangeable working correlation structure was specified for all GEE models. In addition, a separate multivariable GEE logistic regression model was constructed to identify factors associated with AP projection, including age, sex, and radiographic annotations as explanatory variables.

As an additional sensitivity analysis, all primary regression models were repeated after randomly selecting one radiograph per patient. The results of these patient-level analyses were compared with those of the primary image-level analyses to assess the robustness of the observed associations.

All statistical analyses were performed using R version 4.3.2 (R Foundation for Statistical Computing, Vienna, Austria). A two-sided *p*-value < 0.05 was considered statistically significant.

Because the primary analyses were performed at the image level, the reported associations should be interpreted as descriptive dataset-level findings rather than patient-level causal estimates. The additional patient-level GEE analyses were performed to evaluate the potential influence of repeated radiographs on the observed associations.

### 2.5. Sensitivity Analyses

Sensitivity analyses were conducted to evaluate the robustness of the observed associations. Based on previous validation studies of dataset annotations, annotation frequencies and regression analyses were recalculated under simulated misclassification scenarios of ±20%. In addition, a patient-level sensitivity analysis restricted to one randomly selected radiograph per patient was performed to confirm that repeated examinations did not materially influence the principal findings.

## 3. Results

A total of 4988 pneumothorax and 5588 pulmonary mass annotations were identified within the dataset. Pneumothorax was distributed almost equally between males (*n* = 2512) and females (*n* = 2476). Female patients with pneumothorax were significantly older than male patients (49.4 ± 14.5 vs. 47.7 ± 16.4 years, *p* < 0.0001). In addition, projection type differed significantly according to sex, with female patients more frequently undergoing posteroanterior (PA) imaging (*p* < 0.0001) (Table 1).

Pulmonary mass annotations were more frequently detected in male patients than in female patients. Significant age- and sex-related differences were observed across several age categories. Detection frequencies varied according to radiographic projection in the primary image-level analyses; however, the association between AP projection and pulmonary mass annotations was attenuated and no longer statistically significant in the corresponding patient-level GEE analyses.

**Figure 1:**
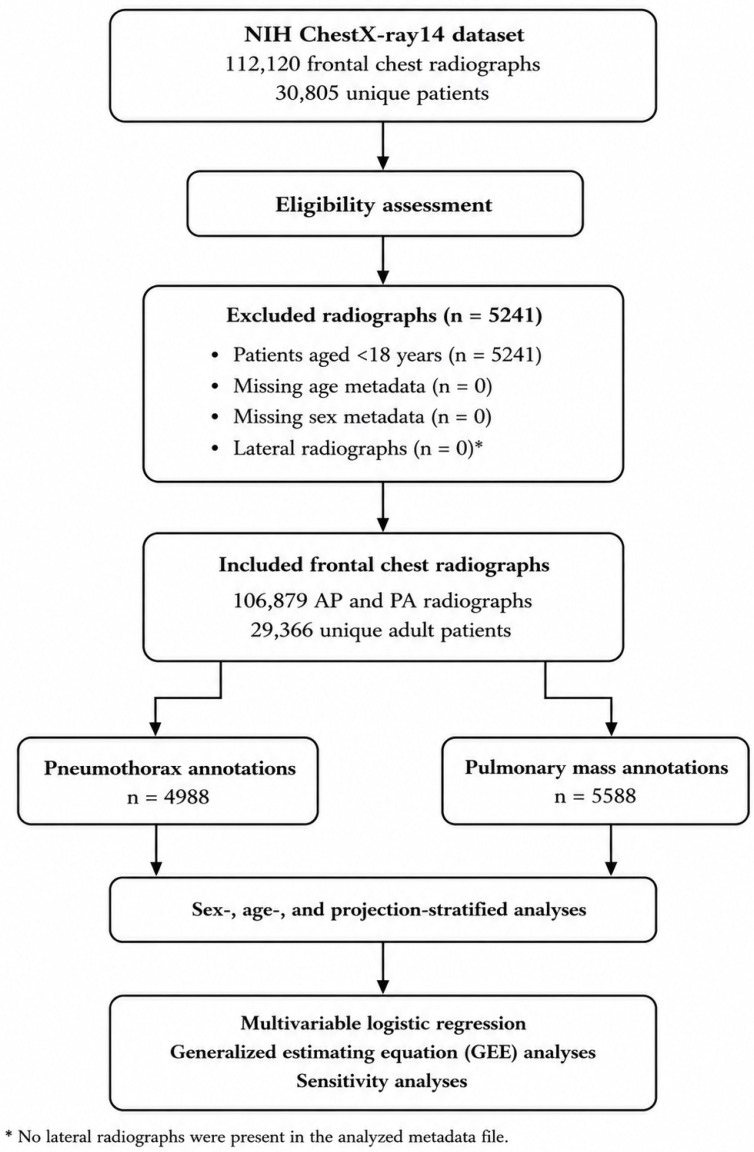
Flowchart illustrating the study selection process, eligibility assessment, and analytical workflow. A total of 112,120 frontal chest radiographs from 30,805 unique patients in the NIH ChestX-ray14 dataset were screened according to the predefined inclusion and exclusion criteria. After excluding radiographs from patients younger than 18 years, 106,879 frontal AP and PA radiographs from 29,366 unique adult patients were included in the final analysis. Pneumothorax and pulmonary mass annotations were identified from the dataset labels, followed by image-level analyses, additional patient-level generalized estimating equation (GEE) analyses accounting for repeated radiographs, and sensitivity analyses.

**Figure 2:**
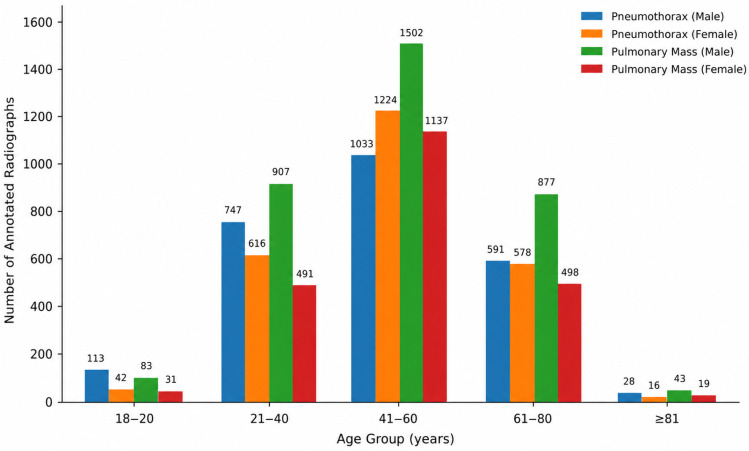
Age- and sex-specific distribution of radiographs annotated as pneumothorax and pulmonary mass. Bars represent the image-level numbers of radiographs annotated as pneumothorax or pulmonary mass according to age group and sex. Pulmonary mass annotations were more common among males across most age groups, whereas pneumothorax annotations were more common among females aged 41–80 years. The figure presents image-level annotation counts rather than disease prevalence because the annotations were generated from radiology reports using natural language processing (NLP) within the NIH ChestX-ray14 dataset.

The study selection process and exclusion criteria are summarized in Figure 1. The flowchart additionally reports the number of excluded radiographs for each exclusion criterion and the final numbers of included radiographs and unique patients. Figure 2 illustrates the age- and sex-specific distribution of pneumothorax and pulmonary mass annotations across the predefined age groups. Detailed demographic characteristics, age-stratified analyses, and multivariable regression results are shown in Table 1, Table 2 and Table 3.

Additional patient-level analyses using generalized estimating equation (GEE) logistic regression models were performed to account for repeated radiographs from the same individual. The resulting effect estimates were largely consistent with those obtained in the primary image-level analyses, indicating that the principal findings were robust after accounting for within-patient correlation.

Because the primary analyses were performed at the image level, repeated examinations from the same patient may be represented within the dataset. The consistency between the image-level and patient-level analyses supports the interpretation of the reported associations as robust dataset-level findings. Nevertheless, these findings should not be interpreted as patient-level estimates or measures of disease prevalence because the analyses were based on NLP-derived dataset annotations.

### 3.1. Pneumothorax

Age-stratified analyses demonstrated significant sex-related differences in pneumothorax annotation detection frequencies. Female patients exhibited higher frequencies of pneumothorax annotations within the 41–60 and 61–80-year age groups (adjusted odds ratios [adjusted ORs] for males vs. females: 0.68 and 0.66, respectively; both *p* < 0.0001). In contrast, no significant sex-related differences were observed among younger patients (18–40 years) or those aged 81 years and older (Table 2, Figure 2).

These results indicate age-dependent variation in the distribution of pneumothorax annotations across demographic subgroups. Additional patient-level generalized estimating equation (GEE) analyses accounting for repeated radiographs from the same individual yielded effect estimates comparable to those obtained in the primary image-level analyses, indicating that repeated examinations did not materially influence the observed associations. Because pneumothorax annotations were derived from NLP-generated annotations in the NIH ChestX-ray14 dataset rather than clinically confirmed diagnoses, the results should be interpreted as dataset-level associations rather than estimates of disease prevalence, patient-level risk, or causal relationships.

In multivariable generalized estimating equation (GEE) logistic regression analyses, male sex (adjusted OR 0.81; 95% CI 0.76–0.85; *p* < 0.0001) and anteroposterior (AP) projection (adjusted OR 0.83; 95% CI 0.79–0.88; *p* < 0.0001) were independently associated with lower odds of pneumothorax annotation detection. Increasing age demonstrated a borderline association with pneumothorax annotation detection frequency (adjusted OR 0.998 per year; 95% CI 0.996–1.000; *p* = 0.059) (Table 3). Additional patient-level analyses using generalized estimating equation (GEE) models, which accounted for repeated radiographs from the same individual, produced comparable effect estimates and confirmed the robustness of these associations. Accordingly, repeated examinations did not materially influence the principal findings. Because the analyses were conducted at the image level, the reported associations should be interpreted as descriptive dataset-level findings rather than patient-level estimates. Furthermore, the reported outcomes reflect NLP-derived dataset annotations and should not be interpreted as expert-confirmed diagnoses.

### 3.2. Pulmonary Mass

Pulmonary mass annotations were more frequently identified in male patients *(n* = 3412) than in female patients (*n* = 2176). No significant difference in mean age was observed between males and females (50.0 ± 16.7 vs. 50.2 ± 14.0 years; *p* = 0.59). In patient-level generalized estimating equation (GEE) analyses accounting for repeated radiographs from the same individual, male sex remained independently associated with higher odds of pulmonary mass annotation detection (adjusted OR 1.22; 95% CI 1.08–1.37; *p* = 0.001) (Table 3).

Age-stratified analyses demonstrated significant sex-related differences in pulmonary mass annotation detection frequencies. The strongest association was observed in the 21–40-year age group (adjusted OR 1.52; *p* < 0.0001). Significant differences also remained evident among patients aged 41–60 and 61–80 years, whereas no significant sex-related differences were identified in the youngest (18–20 years) or oldest (≥81 years) age groups (Table 4, Figure 2).

Patient-level GEE analyses yielded effect estimates comparable to those obtained in the primary image-level analyses, confirming the robustness of the observed associations. Male sex and increasing age remained independently associated with pulmonary mass annotation detection, whereas the association with AP projection was attenuated and was no longer statistically significant after accounting for within-patient correlation.

Because pulmonary mass annotations were derived from NLP-generated annotations in the NIH ChestX-ray14 dataset rather than clinically confirmed diagnoses, the reported associations should be interpreted as dataset-level associations rather than estimates of disease prevalence, patient-level risk, or causal relationships.

In multivariable generalized estimating equation (GEE) logistic regression analyses, male sex (adjusted OR 1.22; 95% CI 1.08–1.37; *p* < 0.001) and increasing age (adjusted OR 1.006 per year; 95% CI 1.003–1.010; *p* < 0.001) remained independently associated with higher odds of pulmonary mass annotation detection. In contrast, the association between anteroposterior (AP) projection and pulmonary mass annotation detection was attenuated and was no longer statistically significant after accounting for repeated radiographs from the same individual (adjusted OR 0.93; 95% CI 0.85–1.01; *p* = 0.097) (Table 3). The similarity between the image-level and patient-level analyses indicates that repeated examinations did not materially influence the principal findings. Because the reported outcomes were derived from NIH ChestX-ray14 dataset annotations generated from radiology reports using natural language processing, the observed associations should be interpreted as dataset-level associations rather than clinically confirmed diagnoses or estimates of disease prevalence.

### 3.3. Predictors of Projection Type

In a separate generalized estimating equation (GEE) logistic regression model, male sex (adjusted OR 1.15; 95% CI 1.13–1.18; *p* < 0.0001) and younger age (adjusted OR 0.996 per year; 95% CI 0.996–0.997; *p* < 0.0001) were independently associated with increased odds of AP imaging. Conversely, radiographs annotated as pneumothorax (adjusted OR 0.83; 95% CI 0.79–0.88; *p* < 0.0001) and radiographs annotated as pulmonary mass (adjusted OR 0.94; 95% CI 0.89–0.99; *p* = 0.017) were associated with reduced odds of AP projection (Table 5).

Because important clinical variables, including patient mobility, disease severity, hospitalization status, intensive care unit admission, smoking history, chronic obstructive pulmonary disease, previous malignancy, and the clinical indication for chest radiography, were not available in the NIH ChestX-ray14 dataset, residual confounding cannot be excluded. Consequently, the observed associations should be interpreted as reflecting both technical imaging characteristics and differences in the underlying clinical context. Furthermore, pneumothorax and pulmonary mass represent NLP-derived dataset annotations rather than reference-standard diagnoses.

## 4. Discussion

In this large-scale analysis of chest radiographs, significant age-, sex-, and projection-related differences in the annotation detection frequencies of pneumothorax and pulmonary mass were identified. These findings highlight the influence of both demographic characteristics and radiographic acquisition techniques on the interpretation of chest radiographs and provide important insights into sources of variability within large imaging datasets. Understanding such variability is relevant for both epidemiological investigations and diagnostic imaging studies involving thoracic diseases.

Although pneumothorax was distributed almost equally between males and females overall, age-stratified analyses revealed notable differences across demographic subgroups. Female patients were older and more frequently underwent posteroanterior (PA) imaging, while higher pneumothorax annotation detection frequencies were observed among women between 41 and 80 years of age. These findings differ from traditional epidemiological studies of spontaneous pneumothorax, which have generally reported a predominance among younger male individuals [11,12,13,14,15]. The discrepancy may reflect differences in patient populations, referral patterns, underlying pulmonary diseases, and imaging utilization characteristics within large radiographic datasets. Furthermore, hospitalized and imaging-intensive populations may exhibit demographic distributions that differ substantially from those observed in population-based cohorts. Because the analyses were based on NLP-derived NIH ChestX-ray14 dataset annotations rather than clinically confirmed diagnoses, these findings should be interpreted as reflecting the distribution of annotated radiographic findings within the dataset rather than the true epidemiology of pneumothorax.

Multivariable analyses demonstrated that male sex and anteroposterior (AP) projection were independently associated with lower odds of pneumothorax annotation detection. These observations are consistent with the known technical limitations of AP imaging, particularly in supine, critically ill, or immobilized patients, in whom subtle pleural abnormalities may be more difficult to identify [12,13]. Because variables such as patient mobility, disease severity, hospitalization status, and intensive care unit admission were unavailable within the dataset, the observed associations should not be interpreted as purely technical acquisition effects. Instead, they likely reflect a combination of imaging-related and clinical factors that influence both projection selection and annotation visibility.

Pulmonary mass annotations were more frequently identified in male patients across most age groups, particularly among younger and middle-aged adults. The observed distribution of pulmonary mass annotations broadly parallels previously reported demographic patterns of thoracic malignancies and other mass-forming pulmonary diseases [16,17,18]. However, because the present analyses were based on NLP-derived dataset annotations rather than histopathologically confirmed diagnoses, direct epidemiological comparisons should be interpreted with caution. Furthermore, the interpretation and clinical management of pulmonary masses and nodules increasingly rely on standardized radiological definitions and risk-stratification approaches, as reflected in contemporary thoracic imaging and pulmonary nodule guidelines [19,20,21]. In the primary image-level analyses, pulmonary mass annotation detection frequencies were lower in AP projections; however, this association was attenuated and was no longer statistically significant after patient-level generalized estimating equation (GEE) analysis. This observation is consistent with previous studies demonstrating the impact of image acquisition parameters and radiological interpretation variability on the visibility and assessment of thoracic abnormalities [22,23].

Projection type emerged as an important determinant of radiographic findings. Male sex, younger age, and the absence of thoracic abnormalities were associated with increased use of AP imaging. Conversely, pneumothorax and pulmonary mass annotations were less frequently identified on AP radiographs. These observations suggest that projection selection may reflect underlying clinical circumstances and imaging workflows rather than technical preferences alone [23]. Additional patient-level GEE analyses yielded effect estimates comparable to those of the primary image-level analyses, indicating that repeated examinations did not materially influence the principal findings. However, because variables such as emergency imaging, patient mobility, hospitalization status, and intensive care unit admission were not available within the dataset, residual confounding related to clinical context cannot be excluded. Therefore, the observed associations with projection type should not be interpreted as purely technical acquisition effects.

The sensitivity analyses demonstrated that the observed associations remained stable across multiple simulated misclassification scenarios. This finding suggests that the reported age-, sex-, and projection-related differences are unlikely to be explained solely by annotation inaccuracies. The consistency between the primary image-level analyses and the additional patient-level GEE models further supports the robustness of the observed associations. Nevertheless, the use of report-derived annotations rather than independent expert image review remains an important consideration when interpreting the results. Future studies incorporating radiologist-validated annotations with computed tomography as a reference standard may further clarify the magnitude of the observed associations.

Several practical implications arise from these findings. First, demographic characteristics and radiographic acquisition parameters should be considered when interpreting large imaging datasets and when comparing findings across studies. Second, age-, sex-, and projection-specific analyses may improve understanding of variability in thoracic imaging and contribute to more accurate characterization of radiographic annotation patterns. Third, future studies would benefit from the integration of detailed clinical metadata, expert-validated annotations, external validation cohorts, and clinically confirmed outcome measures to improve the reliability and generalizability of imaging-based research.

Large imaging repositories provide valuable opportunities to investigate demographic and technical variability in thoracic imaging and pulmonary disease detection [24,25,26,27]. Consequently, demographic and acquisition-related variability may influence the composition of research datasets and should be considered when interpreting imaging-based epidemiological studies.

Although additional patient-level generalized estimating equation (GEE) analyses confirmed the robustness of the primary image-level findings, the study findings should be interpreted in light of several limitations.

## 5. Limitations

Several limitations should be acknowledged. Although the additional patient-level generalized estimating equation (GEE) analyses demonstrated the robustness of the primary findings, the NIH ChestX-ray14 dataset provides NLP-derived radiographic annotations rather than clinically confirmed diagnoses and lacks important clinical variables, including smoking history, chronic obstructive pulmonary disease, previous malignancy, hospitalization status, intensive care unit admission, disease severity, and the clinical indication for imaging. Consequently, residual confounding cannot be excluded, and the reported associations should be interpreted as descriptive dataset-level findings rather than causal estimates or measures of disease prevalence.

First, radiographic findings were based on NLP-derived annotations extracted from radiology reports rather than independent expert radiologist review. Although previous validation studies have demonstrated reasonable performance for several labels, particularly pneumothorax, annotation accuracy remains imperfect, and misclassification may have influenced the observed associations. While sensitivity analyses were conducted to assess the potential impact of annotation error, independent validation using radiologist-reviewed datasets or computed tomography (CT) as a reference standard would further strengthen the reliability of the findings.

Second, the analysis relied on secondary data and lacked detailed clinical information, including smoking history, chronic obstructive pulmonary disease, previous malignancy, symptom burden, disease severity, hospitalization status, intensive care unit admission, patient mobility, the clinical indication for imaging, and treatment characteristics. These factors may influence both the occurrence of thoracic abnormalities and imaging utilization patterns and therefore represent potential sources of residual confounding. Consequently, the observed associations related to AP and PA imaging should not be interpreted as purely technical acquisition effects.

Third, the study was based on a single publicly available dataset and did not include external validation cohorts. Therefore, the generalizability of the findings to other populations, healthcare systems, or imaging repositories may be limited. Future studies using independent datasets are warranted to confirm the reproducibility and generalizability of the observed associations.

Fourth, although the primary analyses were performed at the image level, additional patient-level GEE analyses accounting for repeated radiographs from the same individual yielded comparable effect estimates, indicating that repeated examinations did not materially influence the principal conclusions of the study. Nevertheless, the primary analyses remain descriptive because the dataset contains repeated radiographic examinations rather than independent patient observations.

Finally, although the NIH ChestX-ray14 dataset has been widely used in imaging research, variability in annotation quality, patient population structure, and radiographic acquisition protocols may affect the transferability of the findings to other datasets. Nevertheless, the large sample size, the consistency between the primary image-level analyses and the additional patient-level GEE analyses, and the systematic analytical approach provide valuable insights into demographic and technical sources of variability in chest radiograph interpretation.

## 6. Conclusions

This study identified significant age-, sex-, and projection-related differences in the annotation detection frequencies of pneumothorax and pulmonary mass within a large chest radiograph dataset. Pneumothorax annotations were more frequently identified among females in middle-aged and older age groups, whereas pulmonary mass annotations were more commonly observed in males across most age categories. In addition, anteroposterior projection remained independently associated with lower pneumothorax annotation detection frequencies, whereas its association with pulmonary mass annotations was attenuated and no longer statistically significant after patient-level GEE analyses.

These findings demonstrate that demographic characteristics and imaging acquisition parameters represent important sources of variability in chest radiograph interpretation. Consideration of such factors may improve the interpretation of large imaging datasets and contribute to more accurate imaging-based epidemiological and diagnostic research involving thoracic diseases.

The use of NLP-derived dataset annotations, the absence of detailed clinical information, and the lack of external validation cohorts remain important limitations and underscore the need for future studies incorporating expert-validated labels, comprehensive clinical metadata, and independent validation datasets. Although the primary analyses were performed at the image level, additional patient-level generalized estimating equation (GEE) analyses accounting for repeated radiographs from the same individual yielded comparable effect estimates, supporting the robustness of the principal findings.

Overall, this study demonstrates that age, sex, and radiographic projection significantly influence the distribution of pneumothorax and pulmonary mass annotations within a large chest radiograph dataset. Although these findings should be interpreted as dataset-level associations rather than clinically confirmed diagnoses or estimates of disease prevalence, they highlight the importance of considering demographic characteristics and radiographic acquisition parameters when interpreting large-scale chest radiograph datasets and conducting imaging-based epidemiological and diagnostic studies.

## Figures and Tables

**Table 1:** Demographic and imaging characteristics of pneumothorax and pulmonary mass annotations according to sex. Data are presented as the number of cases, mean age (±standard deviation), and distribution of radiographic projections (anteroposterior [AP] and posteroanterior [PA]). *p*-values indicate statistical significance for differences between male and female patients. The primary descriptive analyses were performed at the image level. Additional patient-level analyses accounting for repeated radiographs from the same individual yielded comparable results, supporting the robustness of the observed associations. The reported findings are based on NIH ChestX-ray14 dataset annotations and should not be interpreted as independently verified diagnoses.

Variable	Male	Female	*p*-Value
Pneumothorax			
Cases, *n*	2512	2476	—
Age, years (mean ± SD)	47.7 ± 16.4	49.4 ± 14.5	<0.0001
AP projection, *n* (%)	1025 (40.8)	694 (28.0)	<0.0001
PA projection, *n* (%)	1487 (59.2)	1782 (72.0)	<0.0001
Pulmonary mass			
Cases, *n*	3412	2176	—
Age, years (mean ± SD)	50.0 ± 16.7	50.2 ± 14.0	0.5944
AP projection, *n* (%)	1348 (39.5)	772 (35.5)	0.0021 *
PA projection, *n* (%)	2064 (60.5)	1404 (64.5)	0.0021 *

* Calculated from chi-square comparison of projection distribution between sexes. Descriptive statistics are presented at the image level. Additional patient-level generalized estimating equation (GEE) analyses accounting for repeated radiographs yielded effect estimates comparable to those obtained in the primary image-level analyses, confirming the robustness of the observed associations.

**Table 2:** Data are presented as the number of pneumothorax-positive radiographs and the total number of radiographs (*n*/*N*) among male and female patients across age groups. Adjusted odds ratios (adjusted ORs) comparing pneumothorax annotation detection frequencies between males and females are reported with corresponding 95% confidence intervals (CIs) and *p*-values. Multivariable logistic regression analyses were adjusted for radiographic projection (anteroposterior versus posteroanterior). Pneumothorax represents the corresponding NIH ChestX-ray14 dataset annotation derived from radiology reports using natural language processing and should not be interpreted as a clinically confirmed diagnosis.

Age Group (years)	Male Cases/ Total (%)	Female Cases/ Total (%)	Adjusted OR (Male vs. Female)	95% CI	*p*-Value
18–20	113/1583 (7.1)	42/988 (4.3)	1.81	1.26–2.61	0.0014
21–40	747/16,655 (4.5)	616/13,488 (4.6)	0.99	0.89–1.10	0.828
41–60	1033/27,233 (3.8)	1224/22,269 (5.5)	0.69	0.63–0.75	<0.0001
61–80	591/14,357 (4.1)	578/9409 (6.1)	0.65	0.58–0.74	<0.0001
≥81	28/499 (5.6)	16/392 (4.1)	1.36	0.72–2.56	0.337

Notes. Additional patient-level generalized estimating equation (GEE) analyses accounting for repeated radiographs from the same patient yielded comparable effect estimates, supporting the robustness of the primary image-level findings.

**Table 3:** Multivariable generalized estimating equation (GEE) logistic regression analyses of factors associated with pneumothorax and pulmonary mass annotation detection. Adjusted odds ratios (adjusted ORs) and corresponding 95% confidence intervals (CIs) are presented for sex, age, and radiographic projection (anteroposterior [AP] versus posteroanterior [PA]). To account for repeated radiographs from the same individual, additional patient-level generalized estimating equation (GEE) logistic regression analyses were performed using patient ID as the clustering variable. The patient-level analyses yielded comparable effect estimates, confirming the robustness of the primary image-level findings. Pneumothorax and pulmonary mass represent NIH ChestX-ray14 dataset annotations derived from radiology reports using natural language processing rather than clinically confirmed diagnoses.

Outcome	Variable	Adjusted OR * (GEE)	95% CI	*p*-Value
Pneumothorax annotation	Male sex	0.779	0.661–0.916	0.0026
	Age (per year)	1.000	0.994–1.005	0.9349
	AP projection (vs. PA)	0.803	0.704–0.916	0.0011
Pulmonary mass annotation	Male sex	1.219	1.084–1.371	0.0010
	Age (per year)	1.006	1.003–1.010	0.0005
	AP projection (vs. PA)	0.929	0.851–1.013	0.0966

* Adjusted odds ratios were estimated using GEE models with patient ID as the clustering variable.

**Table 4:** Sex-specific differences in pulmonary mass annotation detection frequencies according to age group (≥18 years). Data are presented as the number of radiographs annotated as pulmonary mass and the total number of radiographs (*n*/*N*) among male and female patients across age groups. Adjusted odds ratios (adjusted ORs) comparing pulmonary mass annotation detection frequencies between males and females are reported with corresponding 95% confidence intervals (CIs) and *p*-values. Multivariable logistic regression analyses were adjusted for radiographic projection (anteroposterior versus posteroanterior).

Age Group (years)	Male Cases/ Total (%)	Female Cases/ Total (%)	Adjusted OR (Male vs. Female)	95% CI	*p*-Value
18–20	83/1583 (5.2)	31/988 (3.1)	1.79	1.17–2.73	0.0070
21–40	907/16,655 (5.4)	491/13,488 (3.6)	1.53	1.37–1.72	<0.0001
41–60	1502/27,233 (5.5)	1137/22,269 (5.1)	1.08	1.00–1.17	0.0450
61–80	877/14,357 (6.1)	498/9409 (5.3)	1.16	1.04–1.30	0.0101
≥81	43/500 (8.6)	19/392 (4.8)	1.81	1.03–3.16	0.0389

Notes. Additional patient-level generalized estimating equation (GEE) analyses accounting for repeated radiographs from the same patient yielded comparable effect estimates, supporting the robustness of the primary image-level findings.

**Table 5:** Multivariable generalized estimating equation (GEE) logistic regression analysis of factors associated with anteroposterior (AP) projection. Adjusted odds ratios (adjusted ORs) and corresponding 95% confidence intervals (CIs) are presented for sex, age, and radiographic annotations. Patient ID was specified as the clustering variable to account for repeated radiographs from the same individual. Pneumothorax and pulmonary mass represent NIH ChestX-ray14 dataset annotations derived from radiology reports using natural language processing rather than clinically confirmed diagnoses.

Variable	Adjusted OR (GEE)	95% CI	*p*-Value
Male sex	1.15	1.12–1.17	<0.0001
Age (per year)	0.996	0.995–0.997	<0.0001
Pneumothorax annotation	0.83	0.79–0.88	<0.0001
Pulmonary mass annotation	0.94	0.89–0.99	0.017

Notes. Adjusted odds ratios (adjusted ORs) were estimated using generalized estimating equation (GEE) logistic regression models, with patient ID specified as the clustering variable to account for repeated radiographs from the same individual. Pneumothorax and pulmonary mass refer to NLP-derived annotations in the NIH ChestX-ray14 dataset and should not be interpreted as clinically confirmed diagnoses.

## Data Availability

The NIH ChestX-ray14 dataset analyzed in this study is publicly available through the National Institutes of Health (NIH) repository. All analyses were performed using this publicly accessible dataset.
